# CT ventilation image-guided helical Tomotherapy at sparing functional lungs for locally advanced lung cancer: analysis of dose-function metrics and the impact on pulmonary toxicity

**DOI:** 10.1186/s13014-022-02189-x

**Published:** 2023-01-09

**Authors:** Shuangshuang Li, Juan Liu, Shanbao Gao, Yicai Yin, Ling Zhang, Yongchao Han, Xishun Zhang, Yuanyuan Li, Jing Yan, Zhen Hou

**Affiliations:** The Comprehensive Cancer Centre of Nanjing Drum Tower Hospital, The Affiliated Hospital of Nanjing University Medical School, Nanjing, 210000 Jiangsu China

**Keywords:** 4DCT, Pulmonary ventilation, Intensity-modulated radiotherapy, Helical Tomotherapy, Lung cancer

## Abstract

**Purpose:**

CT ventilation image (CTVI)-guided radiotherapy that selectively avoids irradiating highly-functional lung regions has potential to reduce pulmonary toxicity. Considering Helical TomoTherapy (HT) has higher modulation capabilities, we investigated the capability and characteristic of HT at sparing functional lungs for locally advanced lung cancer.

**Methods and materials:**

Pretreatment 4DCT scans were carried out for 17 patients. Local lung volume expansion (or contraction) during inspiration is related to the volume change at a given lung voxel and is used as a surrogate for ventilation. The ventilation maps were generated from two sets of CT images (peak-exhale and peak-inhale) by deformable registration and a Jacobian-based algorithm. Each ventilation map was normalized to percentile images. Six plans were designed for each patient: one anatomical plan without ventilation map and five functional plans incorporating ventilation map which designed to spare varying degrees of high-functional lungs that were defined as the top 10%, 20%, 30%, 40%, and 50% of the percentile ventilation ranges, respectively. The dosimetric and evaluation factors were recorded regarding planning target volume (PTV) and other organs at risk (OARs), with particular attention to the dose delivered to total lung and functional lungs. An established dose-function-based normal tissue complication probability (NTCP) model was used to estimate risk of radiation pneumonitis (RP) for each scenario.

**Results:**

Patients were divided into a benefit group (8 patients) and a non-benefit group (9 patients) based on whether the RP-risk of functional plan was lower than that of anatomical plan. The distance between high-ventilated region and PTV, as well as tumor volume had significant differences between the two groups (*P* < 0.05). For patients in the benefit group, the mean value of fV5, fV10, fV20, and fMLD (functional V5, V10, V20, and mean lung dose, respectively) were significantly lower starting from top 30% functional plan than in anatomical plan (*P* < 0.05). With expand of avoidance region in functional plans, the dose coverage of PTV is not sacrificed (*P* > 0.05) but at the cost of increased dose received by OARs.

**Conclusion:**

Ventilation image-guided HT plans can reduce the dose received by highly-functional lung regions with a range up to top 50% ventilated area. The spatial distribution of ventilation and tumor size were critical factors to better select patients who could benefit from the functional plan.

**Supplementary Information:**

The online version contains supplementary material available at 10.1186/s13014-022-02189-x.

## Background

Radiotherapy (RT) plays an efficient role in curative treatment of inoperable lung cancer, particularly for locally advanced disease. However, radiation-induced lung injury has still been observed and is associated with radiation pneumonitis (RP), which can affect the prognosis for patients and the quality of life during and after RT. Although intensity-modulated radiotherapy (IMRT) has been shown to reduce the risk of developing RP without compromising tumor-dose coverage [1], yet it is reported that up to 30% of lung cancer patients still occurred with RP after RT [2, 3].

Currently, the widely-accepted definition of organ at risk (OAR) in radiotherapy planning assumes the entire lung tissue works equally, irrespective of function [4]. However, prior works have documented that functional heterogeneity within the lung could be further changed as the disease progresses [5]. This has then led to the application of lung functional imaging for radiotherapy planning. The functional image-guided radiotherapy planning for normal lung avoidance may reduce lung toxicity and has been incorporated with several imaging modalities [6]: primarily single photon-emission computed tomography (SPECT) [7, 8], but also hyperpolarized helium-3 magnetic resonance imaging (^3^He-MRI) [9, 10]. However, there are still some limitations with respect to the low resolution of SPECT, the need for tracer gas of MR imaging, and the trade-offs between monetary cost and effectiveness, all of which prevent their routine use in the clinic.

To avoid using SPECT or MRI, a new form of lung function imaging has been developed, using four-dimensional CT (4DCT) along with image processing techniques to generate lung ventilation maps, namely CT-ventilation [11]. Then, lung function information gained from the CT ventilation was incorporated into treatment plan optimization. The additional benefit of using CT-ventilation for treatment planning is that the functional information was available from routinely acquired 4DCT, with higher resolution and lower cost, without subjecting patients to extra radiation exposure or imaging [12]. Recently, researchers have determined the effectiveness of CT ventilation-guided radiotherapy planning for functional lung avoidance using photon beams, including three-dimensional conformal radiotherapy (3D-CRT) [13] and intensity-modulated radiotherapy (IMRT) [8, 14, 15]. However, to our knowledge, there is no established threshold to quantify absolute lung function with a CT-ventilation map, the top 10%—top 30% of the percentile ventilation ranges were considered as the high functional lung in recent studies [13–16]. Studies of sparing wider ventilation zones (e.g., top40% and top50%) have been rarely reported. Furthermore, because the complex geometrical constraints were generated by the ventilation map, it is challenging to optimize beam angles and field numbers manually in static IMRT planning processes [14]. Compared with standard 7- to 9-field IMRT plans manually select beam angles, Helical Tomotherapy (HT) has higher modulation capabilities with 51 fields per rotation and therefore provides more degrees of freedom in beam arrangement for inverse optimization involving complex constrained structures [17].

This work aimed to analyze dose-function metrics and the impact on pulmonary toxicity of CT ventilation functional image-guided HT treatment planning at sparing various lung function regions (top10% to top50%) for locally advanced lung cancer.

## Materials and methods

### Patient characteristics

Seventeen patients with non-small cell lung cancer who previously underwent Helical TomoTherapy (HT) at Nanjing Drum Tower Hospital were selected. Pretreatment 4DCT data were obtained for all patients to determine the internal margin of the clinical. Patient characteristics are summarized in Table 1. Planning CT and 4DCT scans were performed using a Siemens SOMATOM Definition CT scanner (Siemens, Erlangen, Germany) with a 5 mm slice thickness covering the entire chest. The following standard scan parameters were used: 120 kVp, 120 mA, 0.80.8 pixel spacing.

**Table 1:** Baseline characteristics of 17 patients with locally advanced lung cancer

Characteristic	Value
*Sex*	
Male	14
Female	3
*Age (years)*	
Median (range)	67 (47–89)
*Stage*	
III	5
IV	12
*Tumor location*	
Right upper lobe	5
Right lower lobe	4
Left upper lobe	6
Left lower lobe	2
PTV volume (*cm*^*3*^)	
Median (range)	167.8 (38.24–396.89)

### 4DCT-based ventilation imaging

**Fig. 1:**
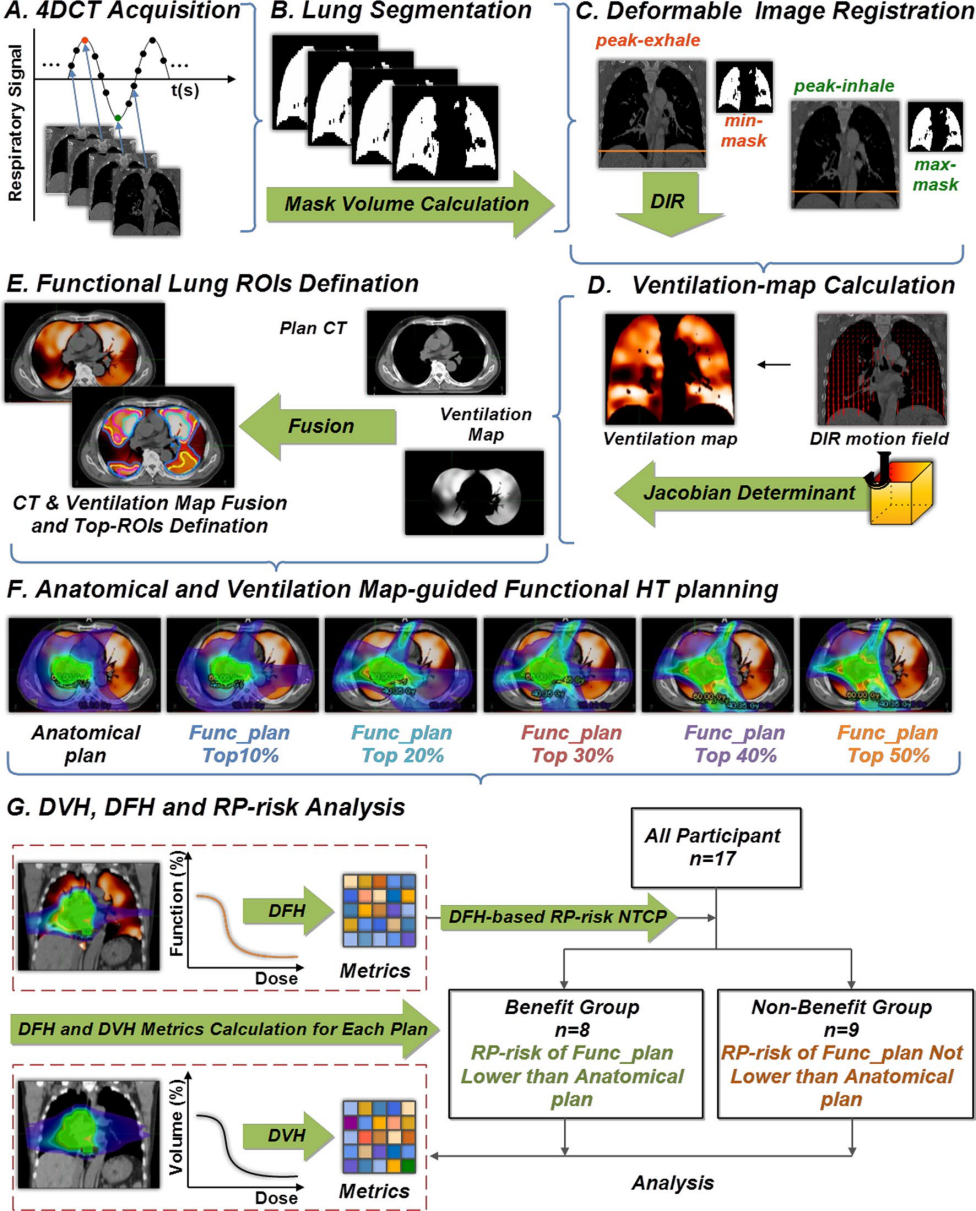
Flowchart of ventilation-map guided HT plan in this work. **A** 4DCT acquisition; **B** Lung segmentation; **C** Deformable Image Registration; (**D**) Ventilation map calculation; **E** Functional lung ROIs definition; **F** Anatomical and ventilation map-guided functional HT plan; **G** Dose-volume histogram (DVH), dose-function histogram (DFH) and RP-risk analysis

All 4D-CT ventilation images (CTVI) were created using VESPIR (VEntilation via Scripted Pulmonary Image Registration), an open-source CTVI toolkit [18]. As shown in Fig. 1, the method contains the five steps: (1) lung segmentation was performed for each phase 4DCT, (2) peak-exhale and peak-inhale phase images were then automatically identified according to the lung mask volume, (3) B-spline elastic algorithm was performed for deformable image registration (DIR) between the above two phase images, (4) Jacobian determinant of the DIR motion field was used as a surrogate for ventilation, and (5) each ventilation map was normalized by converting it to percentile image. The Jacobian-based regional $V_{Jac} (x,y,z)$ ventilation was given in Eq. 1:

1 $${\text{V}}_{Jac} (x,y,z) = \left| {\begin{array}{*{20}c} {1 + \frac{{\partial u_{x} (x,y,z)}}{\partial x}} & {\frac{{\partial u_{x} (x,y,z)}}{\partial y}} & {\frac{{\partial u_{x} (x,y,z)}}{\partial z}} \\ {\frac{{\partial u_{y} (x,y,z)}}{\partial x}} & {1 + \frac{{\partial u_{y} (x,y,z)}}{\partial y}} & {\frac{{\partial u_{y} (x,y,z)}}{\partial z}} \\ {\frac{{\partial u_{z} (x,y,z)}}{\partial x}} & {\frac{{\partial u_{z} (x,y,z)}}{\partial y}} & {1 + \frac{{\partial u_{z} (x,y,z)}}{\partial z}} \\ \end{array} } \right| - 1$$

The equation represents the DIR motion field of each voxel between the peak-exhale image and peak-inhale image. Positive (or negative) values of $V_{Jac} (x,y,z)$ indicate local lung volume expansion (or contraction) during inspiration, which is used as a surrogate for ventilation. Prior studies have shown that CTVI has good regional accuracy with Galligas PET [19] and hyperpolarized gas MRI [20], and has been incorporated to guide functional-avoidance planning [8, 13–15].

### Ventilation image-guided HT treatment planning

For each patient, MIM Meastro software (MIM Software, Inc., Cleveland, OH, United States) was employed to delineate the internal gross tumor volume (IGTV) on 4D-CT and then transfer the contours to planning CT. A clinical target volume (CTV) was subsequently created by adding a 5 mm margin isotropically and was then expanded by 5 mm in all directions to generate the planning target volume (PTV) for setup uncertainty. The organs at risk (OARs), including the spinal cord, esophagus, heart, and total lungs, were delineated on planning CT.

Because there is no established threshold, to our knowledge, to quantify absolute lung function with a CT-ventilation map, the top 10%, top 20%, top 30%, top 40%, and top 50% areas of the percentile ventilation ranges were respectively considered in this work. The corresponding regions of interest (ROIs) were generated according to the different thresholds using MIM Meastro and were included in the functional contours used in the planning (Fig. 1).

For each patient, one anatomical plan without incorporating the ventilation map and five functional plans with ventilation information (sparing top 10%, top 20%, top 30%, top 40%, and top 50% of functional lungs, respectively) were designed using the Helical TomoTherapy (HT) treatment planning system (TomoTherapy, Inc., USA), with the aim of minimizing the dose to functional contours while also attempting to meet Radiation Therapy Oncology Group (RTOG) 0617-based constrains used for acceptable clinical planning (See Additional file 1: Appendix A). The collimator size, pitch and modulation factor used were 2.5 cm, 0.287 and 2.5, respectively. The prescription dose of 60 Gy in 30 fractions was prescribed to cover at least 95% of PTV.

## Dosimetric analysis and pulmonary toxicity assessment

### Structure-based lung dose-volume histogram (DVH) metrics

For the anatomical plan and functional plans of each patient, we calculated the DVH and dosimetric parameters such as mean lung dose (MLD), V5 (percentage of volume receiving dose more than 5 Gy), V10, and V20 were applied to the total lung.

### Ventilation image-based lung dose function histogram (DFH) metrics

The above conventional DVHs do not indicate the dose distributions to functionally heterogeneous normal tissue. In view of this, for various planning strategies, we calculated the functional dose metrics (fV5, fV10, fV20, fMLD) from the dose-function histogram (DFH), which relates the dose to the fraction of total lung ventilation function value at that dose.

### Pulmonary toxicity calculation

To assess how much improvement in dosimetry can translate into a reduction in the probability of pulmonary toxicity, the probabilities of grade 2 + radiation pneumonitis (RP) were calculated using a DFH-based normal tissue complication probability (NTCP) models proposed by Faught et al. [21]. The NTCP models were fit by DFH metrics using the maximum-likelihood method. In this work, we employed the fitting parameters of models which showed the best performance for grade 2 + RP (fV10, *m* = 0.53, TD_50_ = 54.1).

Patients were divided into two groups based on whether patients’ pulmonary toxicity benefits from the functional plan, as described below:

In group 1 (RP-risk benefit group), patients’ RP risk of the functional plan was lower compared with the anatomical plan; in group 2 (RP-risk non-benefit group), patients’ RP risk of the functional plan was higher than that of the anatomical plan.

### Dosimetric parameters of PTV as well as other critical structures

For each plan, we also calculated the homogeneity index (HI) and conformity index (CI), representing dose uniformity and treatment volume ratio of PTV, to evaluate the acceptability of the anatomical and functional plan. To quantify the distances between PTV and highly-functional lung regions, the Hausdorff distances (HD) between the PTV and functional structures (top10% to top50%) were computed for each patient.

For adjacent organ of risk (OAR), dosimetric parameters of the heart (D_mean_, V45), esophagus (D_mean_), and spinal cord (D_max_) were also recorded.

### Statistical analysis

For the two groups (RP-risk benefit group *vs.* RP-risk non-benefit group), the HD metrics were investigated to examine whether the distance between PTV and functional lung structures was significantly different. For the RP-risk benefit group (Group 1), the dose metrics (DVH and DFH) of anatomical plans were compared with those of the functional plans for each scenario separately (anatomic plan *vs.* functional top10%/20%/30%/40%/50% plan) to test whether the difference was statistically significant (*P* < 0.05). The paired two-tailed *t*-tests were performed for the metrics that were found to meet the criteria for normality. When the metrics did not meet normality criteria, a two-tailed Wilcoxon rank-sum was used.

## Results

### Patient grouping according to whether RP-risk benefited from the functional plan

All anatomical and functional plans generated in this study were clinically acceptable. According to whether the RP-risk of the functional plan was lower than that of the anatomical plan, eight patients were divided into the benefit group (Group1) and nine patients into the non-benefit group (Group 2). A summary of toxicity probability for different planning strategies for the two groups is presented in Table 2 (Group 1) and Table 3 (Group 2), respectively.

**Table 2:** Summary of ROI-based lung DVH metrics, functional map-based DFH metrics and DFH metrics-based Normal tissue complication probabilities (NTCP) for grade 2 + radiation pneumonitis (RP) among RP-risk benefit patients (Group 1)

Variable	Planning strategy					
	Anatomical plan	Func_ plan _Top10_	Func_ plan _Top20_	Func_ plan _Top30_	Func_ plan _Top40_	Func_ plan _Top50_
DFH metric-based NTCP model (toxicity probability of the mean)						
NTCP_fV10_(%)	38.37 ± 17.14	44.63 ± 21.83	40.84 ± 22.01	33.82 ± 17.35	30.98 ± 15.76^*^	29.77 ± 15.46^**^
Ventilation map-based lung DFH metrics (mean values)						
fV5 (%)	63.94 ± 16.45	68.19 ± 16.29	64.21 ± 15.86	57.66 ± 15.70^*^	54.70 ± 18.36^*^	52.97 ± 17.18^**^
fV10(%)	44.69 ± 13.77	49.77 ± 17.22	46.45 ± 18.00	40.26 ± 15.74	37.77 ± 15.40^*^	36.86 ± 14.96^*^
fV20 (%)	26.41 ± 12.48	24.84 ± 12.24	25.21 ± 12.41	24.05 ± 11.70^*^	23.20 ± 11.55^*^	23.06 ± 11.57^*^
fMLD (Gy)	8.12 ± 2.96	8.17 ± 2.91	8.06 ± 3.04	7.64 ± 3.04^*^	7.55 ± 3.23^*^	7.44 ± 3.20^*^
Structure-based lung DVH metrics (mean values)						
V5 (%)	62.32 ± 12.34	66.09 ± 12.53	63.11 ± 11.60	57.76 ± 11.02^*^	55.15 ± 12.94^**^	53.51 ± 11.85^**^
V10(%)	43.24 ± 9.77	49.87 ± 12.88	47.60 ± 12.94	41.45 ± 11.04	38.62 ± 10.82	37.38 ± 10.36^*^
V20 (%)	24.47 ± 8.92	24.00 ± 9.41	24.92 ± 9.63	23.70 ± 9.05	22.88 ± 8.78	22.52 ± 8.69
MLD (Gy)	13.13 ± 3.16	13.47 ± 3.21	13.46 ± 3.42	13.18 ± 3.37	11.97 ± 4.26	12.40 ± 3.63

^*^p < 0.05 and **p < 0.01, indicate significantly decreased when compared with anatomical plan; Func_plan _Top10/20/30/40/50_, means top 10%/20%/30%/40%/50% percentile functional volume were defined as the high-functional lung in planning optimization

**Table 3:** Summary of ROI-based lung DVH metrics, functional map-based DFH metrics and DFH metrics-based Normal tissue complication probabilities (NTCP) for grade 2 + radiation pneumonitis (RP) among RP-risk non-benefit patients (Group 2)

Variable	Planning strategy					
	Anatomical plan	Func_ plan _Top10_	Func_ plan _Top20_	Func_ plan _Top30_	Func_ plan _Top40_	Func_ plan _Top50_
DFH metric-based NTCP model (toxicity probability of the mean)						
NTCP_f_ _V10_(%)	20.24 ± 14.68	26.21 ± 20.02	26.81 ± 22.08	23.56 ± 17.25	22.40 ± 16.87	21.47 ± 15.15
Ventilation map-based lung DFH metrics (mean values)						
fV5 (%)	44.26 ± 20.54	52.89 ± 24.78	51.77 ± 25.62	49.96 ± 25.14	46.18 ± 23.29	44.84 ± 21.51
fV10(%)	27.36 ± 13.94	33.21 ± 17.58	33.52 ± 19.05	30.89 ± 15.79	29.77 ± 15.44	29.00 ± 14.46
fV20 (%)	16.72 ± 10.59	17.44 ± 12.55	17.25 ± 11.90	17.09 ± 11.46	16.53 ± 10.97	16.49 ± 10.66
fMLD (Gy)	5.59 ± 2.76	6.13 ± 3.05	6.16 ± 3.23	6.00 ± 3.06	5.82 ± 2.93	5.76 ± 2.82
Structure-based lung DVH metrics (mean values)						
V5 (%)	41.14 ± 16.31	48.81 ± 16.53	47.44 ± 17.12	45.74 ± 16.06	43.12 ± 15.94	42.55 ± 16.42
V10(%)	25.10 ± 9.74	31.38 ± 13.44	31.56 ± 14.09	29.75 ± 11.59	28.15 ± 11.34	27.26 ± 10.29
V20 (%)	14.48 ± 6.90	15.05 ± 6.83	15.63 ± 8.20	15.15 ± 7.80	14.99 ± 8.49	14.81 ± 7.84
MLD (Gy)	8.52 ± 3.30	9.41 ± 3.42	9.54 ± 3.86	9.28 ± 3.66	9.18 ± 3.81	9.06 ± 3.66

^*^p < 0.05 and **p < 0.01, indicate significantly decreased when compared with anatomical plan; Func_plan _Top10/20/30/40/50_, means top 10%/20%/30%/40%/50% percentile functional volume were defined as the high-functional lung in planning optimization

**Fig. 2:**
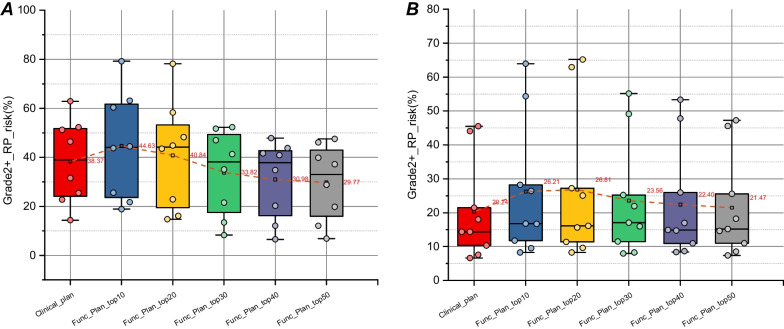
Box plots of mean grade 2 + RP-risk for the benefit group **A** and non-benefit group **B**. Toxicity probabilities are calculated using the dose-function-based NTCP model for 2 + RP-risk as a function of the volume of functional lung receiving 10 Gy (fV10Gy) in anatomical (clinical) and functional plans (Top 10% to 50%)

An illustration of how the variation in toxicity for group 1 and group 2 is shown in Fig. 2 (A) and (B), which shows the mean value of RP-risk for the two groups in different planning strategies. For the benefit group, the risk of Func_plan_Top40_ (Top 40% functional plan) and Func_plan_Top50_ had significantly lower risk compared to anatomical plan with an absolute reduction of 7.39% (*P* < 0.05) and 8.6% (*P* < 0.01) on average, respectively. For the non-benefit group, there was no significant difference in RP-risk between functional versus anatomical plans.

**Fig. 3:**
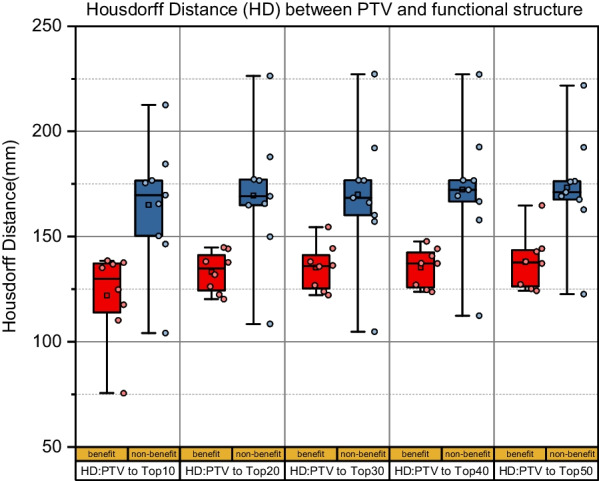
Box plots of mean Hausdorff Distance (HD) between PTV and functional regions for benefit and non-benefit group. The HD values were all found to be significant smaller in the benefit group (red) than in the non-benefit group (blue)

The Hausdorff distance (HD) between the PTV and different functional structures (ventilation of top 10%, 20%, 30%, 40%, and 50%) was calculated for the two groups. The value of HD metrics were all found to be significantly smaller in the benefit group than in the non-benefit group (*P* < 0.05 for all), as shown in Fig. 3. In addition, the differences were again significant for PTV volumes between the two groups (Group 1 *vs.* Group 2, 242.39 ± 103.59 *vs.* 121.64 ± 78.04, *P* < 0.05).

### Comparison of ventilation map-based lung dose-function (DFH) metrics

**Fig. 4:**
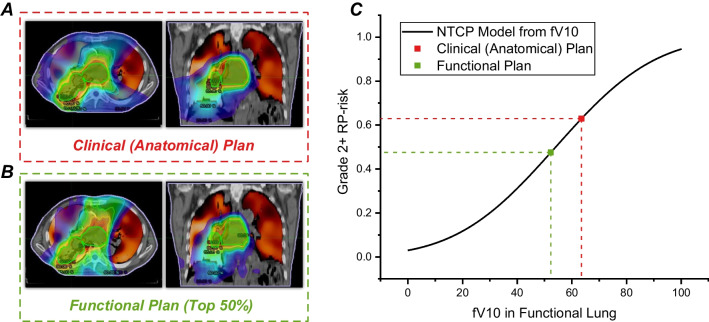
Representative example comparing a clinical (anatomical) plan **A** with a top 50% functional avoidance plan **B**. **C** Illustrated how the reduction in RP-risk was calculated using the dose-function-based NTCP model

The calculated dose-function metrics for each planning strategy, using a lung ventilation map and dose distribution matrix, were computed and compared with the anatomical plan. Table 2 and 3 also show the mean values of DFH metrics for the two groups. For the benefit group, fV5 of Func_plan_Top30/40/50_, fV10 of Func_plan_Top40/50_, fV20 of Func_plan_Top30/40/50_ and fMLD of Func_plan_Top30/40/50_ were found to be significantly lower compared to anatomical plan (*P* < 0.05 or *P* < 0.01). A representative case of a benefit patient with larger reductions in dose to the functional lung is shown in Fig. 4. For the non-benefit group, no statistical differences in DFH parameters were found between the anatomical and functional plans.

### Comparison of structure-based lung dose-volume (DVH) metrics

Functional planning also results in a significant reduction in the lung DVH metrics for the benefit group, for example, V5 by 9.17 Gy (53.51 ± 11.85 Gy *vs.* 62.32 ± 12.34 Gy, *P* < 0.01) and V10 by 5.86 Gy (37.38 ± 10.36 *vs.* 43.24 ± 9.77, *P* < 0.05) on average in Func_plan_Top50_ scenario (Table 2). For the non-benefit group, no significant difference was observed in the lung DVH metrics between the two groups (Table 3).

### Comparison of dose-volume metrics of the PTV and other critical structures

**Fig. 5:**
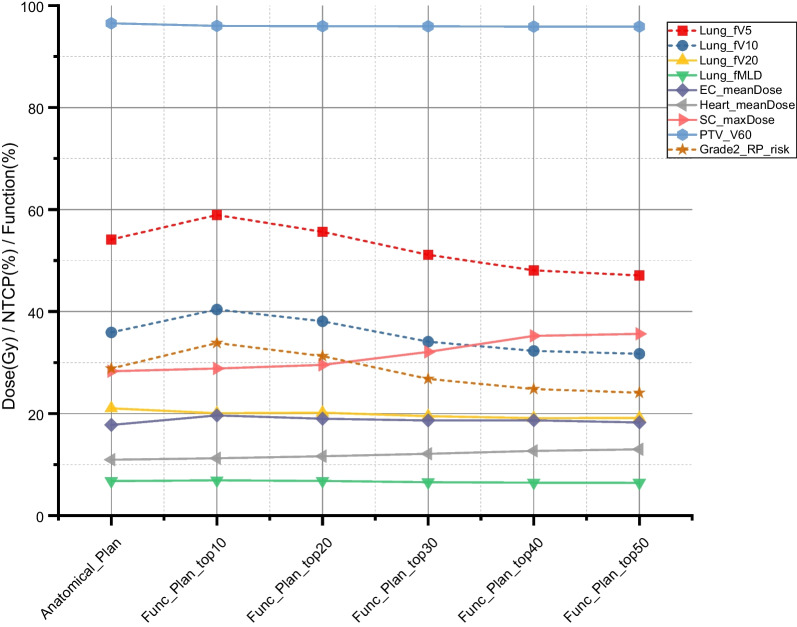
Changes in lung DFH metrics and OARs DVH metrics with expansion of avoidance areas in anatomical and functional plans for the benefit group

The dose to PTV, heart, spinal cord, and esophagus for the benefit group are shown in Table 4. The HI index of PTV was slightly higher for functional plans (Func_plan_Top30/40/50_) compared with the anatomical plan, while the differences of CI index and V60 were not significant. The max dose of the spinal cord (D_max_) and mean dose of the heart (D_mean_) showed an upward trend as the functional avoidance region expanded (Fig. 5). The D_max_ of the spinal cord in Func_plan_Top30/40/50_ showed a significant rise (*P* < 0.05) compared to those in the anatomical plan. Other DVH parameters had no significant differences among the functional planning strategies.

**Table 4:** Comparisons of the mean values of the dosimetric parameters for RP-risk benefit group

Variable	Planning strategy					
	Anatomical plan	Func_plan _Top10_	Func_plan _Top20_	Func_plan _Top30_	Func_plan _Top40_	Func_plan _Top50_
PTV						
V60 (%)	96.47 ± 1.33	96.17 ± 0.47	95.98 ± 0.48	95.98 ± 0.53	95.67 ± 0.57	95.65 ± 0.57
CI	0.85 ± 0.06	0.86 ± 0.03	0.85 ± 0.06	0.84 ± 0.06	0.76 ± 0.11	0.76 ± 0.11
HI	0.06 ± 0.01	0.07 ± 0.01	0.07 ± 0.02	0.09 ± 0.02^**†**^	0.11 ± 0.04^**†**^	0.10 ± 0.03^**†**^
Heart						
D_mean_(Gy)	14.71 ± 6.16	14.56 ± 6.52	15.21 ± 6.74	15.96 ± 7.87	16.74 ± 8.78	16.99 ± 8.64
Spinal cord						
D_max_(Gy)	32.79 ± 5.50	32.16 ± 6.43	33.87 ± 6.37	37.44 ± 6.49^**†**^	40.17 ± 6.89^**†**^	40.54 ± 6.90^**†**^
Esophagus						
D_mean_ (Gy)	22.97 ± 6.70	24.08 ± 6.59	23.88 ± 6.98	23.22 ± 7.13	23.70 ± 7.56	23.49 ± 7.55

^†^*p* < 0.05 and ††*p* < 0.01, indicate significantly increased when compared with anatomical plan; Func_plan _Top10/20/30/40/50_, means top 10%/20%/30%/40%/50% percentile functional volume were defined as the high-functional lung in planning optimization

## Discussion

Understanding the highly-functional lung regions plays a central role in radiotherapy planning optimization and pulmonary toxicity reduction [11, 22, 23]. CT-ventilation has been developed as a form of lung function, using 4DCT images and image processing techniques to provide pulmonary function maps with good regional accuracy with Galligas PET [19] and hyperpolarized gas MRI [20]. Considering the relatively high cost of functional PET and MRI, CT-ventilation-guided functional avoidance radiation treatment has gained momentum in radiotherapy, as patients undergo four-dimensional CT simulation is a standard procedure for lung cancer treatment. Previous literature has reported that 10%—top 30% of the percentile ventilation ranges are generally considered as the high functional lung for planning optimization [13–16]. However, a major issue with the above studies is that protecting a broader region of functional lung (e.g., top40% and top50%) on pulmonary radiotoxicity has not been further investigated. In this study, Helical Tomo (HT) plans were designed to explore how effective the ventilation map-guided HT plan is at sparing various lung function regions for locally advanced lung cancer.

Prior studies have reported that significant dosimetric improvement was observed in the region of the functional lung, while these improvements in reducing the risk of radiation-induced pulmonary toxicity remain unclear [14]. In this study, we translate the reduction in dose to functional lung (as measured by DFH metrics) to a reduction in the risk of RP, using the DFH-based grade 2 + NTCP model proposed by Faught et al. [15]. The result showed that 8 patients (group 1) had RP-risk reduction and the benefit was clear in Func_plan_Top40_ (*P* < 0.05) and Func_plan_Top50_ (P < 0.01), while 9 patients (group2) had no benefit (*P* > 0.05 for all Func_plan). The results suggest that not all patients benefit equally well from a functional avoidance plan, despite the considerable pulmonary toxicity-reducing potential. In addition, a significantly smaller distance (HD) was observed in the benefit group (*P* < 0.05), implying that the PTV was surrounded by highly-functional regions, which may explain why the functional plan has a lower RP risk than the anatomical plan. We also noted that the volume of PTV was larger in the benefit group than in the no-benefit group (*P* < 0.05), which might be another important factor in the ability to profit from a functional avoidance plan. The reason for this might be explained as the beam fields cover less normal lung tissue for small target and therefore has less effect on highly functional ventilation zones. This is consistent with prior studies that have demonstrated the DFH metrics benefit would vary with target size and regional ventilation [16, 24, 25].

Definition of functional lung using a percentile threshold was not consistent throughout publications. In this work, the top 10%, top 20%, top 30%, top 40%, and top 50% area of the percentile ventilation ranges were respectively defined as functional contours for plan optimization. For the benefit group, Fig. 5 displays how the lung DFH metrics change when the functional contour threshold is changed from the top10% percentile to the top50% percentile. The fV5, fV10, fV20, and fMLD were lower than the anatomical plan starting from the top30% functional plan, and the value showed significantly lower in the case of Func_plan_Top40_ and Func_plan_Top50_ (*P* < 0.05). Similar results were also observed in lung DVH metrics (V5, V10, V20, and MLD) which also exhibited a decreasing trend as the size of the avoidance region became larger. In addition, improvements in DFH and DVH only slightly affected dose conformity (CI) and heterogeneity (HI) for PTV but did not compromise the dose coverage. The result suggested that the use of Tomotherapy technique could provide a higher modulated ability to reduce the dose to highly functional ventilation zones, especially for large size of functional structures.

With an increase in the size of the avoidance region (Top10% to Top 50%) in the functional plans, the dose coverage of PTV (*P* > 0.05) is not sacrificed but at the cost of the increased dose received by OARs. During the process of functional lung sparing, there were no significant changes in the mean dose of the esophagus and heart. With the RP_risk and DFH metrics (lung_fV5 and lung_fV10) decreasing, max dose of spinal cord presented two steps: upward region (top10% to top40%) and plateau region (top40% to top50%). A significantly higher max dose of the spinal cord was observed for Func_plan_Top30/40/50,_ which can be attributed to more complex constrained structures in functional planning optimization [16]. Although dose to heart, esophagus, and spinal cord were comparatively higher in functional plans, all OARs satisfied tolerances. Previous literature has reported that field number and beam angle in IMRT may affect the preservation of functional lung [14, 26]. In this work, HT not only reduces the dose to the functional lung but also avoids subjective manual beam setup in static IMRT. Moreover, this work confirms the feasibility of HT in reducing the dose to highly ventilated regions and records the dosimetric characteristic of this process. These findings extend those of O’Reilly et al. [4], which demonstrated that dose to the top45%-60% ventilated regions could improve the prediction of RP risk. Most importantly, based on the results of this study, if a functionally-guided HT plan is to be adopted in practice, clinical decision-making should measure whether the improvement in RP risk can outweigh the disadvantages of dose-escalation of the OARs. This work thus indicates HT radiotherapy could potentially improve the optimization of individualized plans for lung cancer patients, especially for enlarged functional avoidance regions.

Several limitations are worth noting in this study. CT ventilation is an emerging technology with multiple pre-processing steps, including image acquisition, lung segmentation, and deformable image registration, which may introduce uncertainties in calculating ventilation values. Meanwhile, the 3D motion of lung regions is most likely not linearly along the breathing cycle. Better ventilation maps may be generated using all reconstructed phases instead of DIR only the two extremes. Future studies should continue to examine an end-to-end deep learning model to predict the ventilation map based on 4DCT over the entire respiration cycle, minimizing the effects of the 3D nonlinear motion of the lungs and different segmentation or registration algorithms. In addition, the dosimetric parameters and RP risk calculated in this work assume a static spatial distribution between radiation dose and ventilation map, ignoring changes in regional ventilation during treatment. Besides, although the same experienced physicist designed the plans with and without a ventilation map, the planning skills and familiarity degree would gradually refine as time progresses, which may impact dosimetry outcomes. Despite these current limitations and challenges, the promise of functional avoidance planning is immense. This study was valuable in providing evidence of changes in dosimetry parameters and RP-risk for functionally-guided HT plan when avoidance region increased. Future studies, including prospective clinical trials, are needed to explore its clinical significance further.

## Conclusion

Functionally-guided HT plans incorporating CT ventilation images registered to plan CT can reduce the dose received by highly-functional lung regions with a range up to the top 50% of the ventilated area. Our work is the first to explore the changes and characteristics of dose-function metrics and the risk of pulmonary adverse events in functionally-guided HT plan as ventilation avoidance areas expand. The distance between the functional structure and tumor target as well as tumor volume, seem to be critical factors to better select patients who can benefit from the functional plan.

## Supplementary Information

**Additional file 1: Appendix A. **Dose-volume constrains to organs at risk (OAR).

## Data Availability

The datasets used and/or analyzed during the current study are available from the corresponding author on reasonable request.

## Abbreviations

CTVI: CT ventilation image
HT: Helical TomoTherapy
PTV: Planning target volume
OAR: Organs at risk
RP: Radiation pneumonitis
fMLD: Functional mean lung dose
NTCP: Normal tissue complications probability
RT: Radiotherapy
IMRT: Intensity-modulated radiotherapy
IGTV: Internal gross tumor volume
CTV: Clinical target volume
ROI: Regions of interest
DFH: Dose-function histogram
HD: Hausdorff distance
DVH: Dose-volume histogram
CI: Conformity index
HI: Heterogeneity index
