# Supplementary material for: CT ventilation image-guided helical Tomotherapy at sparing functional lungs for locally advanced lung cancer: analysis of dose-function metrics and the impact on pulmonary toxicity
Source: Radiat Oncol. 2023 Jan 9;18:6. doi: 10.1186/s13014-022-02189-x (PMC9830733; doi:10.1186/s13014-022-02189-x)
Supplement: Supplementary file 1 — Additional file 1: Appendix A. Dose-volume constrains to organs at risk (OAR). [file 13014_2022_2189_MOESM1_ESM.docx]

Appendix A. Dose-volume constrains to organs at risk (OAR).

| OAR | Value |
| --- | --- |
| Lung | V_20_ < 37% |
|  | MLD < 20 Gy |
| Spinal cord | Max dose < 47 Gy |
| Esophagus | Max dose < 70Gy |
|  | Mean dose < 34Gy |
| Heart | Max dose < 70 Gy |
|  | V_60_ < 33% |
|  | V_45_ < 66% |

Abbreviations: Vx, percent volume receiving ≥ x Gy; MLD = mean lung dose.
